# Potential therapeutic effects and nano-based delivery systems of mesenchymal stem cells and their isolated exosomes to alleviate acute respiratory distress syndrome caused by COVID-19

**DOI:** 10.1016/j.reth.2024.03.015

**Published:** 2024-04-16

**Authors:** Mohsen Ghiasi, Peyman Kheirandish Zarandi, Abdolreza Dayani, Ali Salimi, Ehsan Shokri

**Affiliations:** a Rajaie Cardiovascular Medical and Research Center, Iran University of Medical Sciences, Tehran, Iran; b Department of Biology, Science and Research Branch, Islamic Azad University, Tehran, Iran; c Nanobiotechnology Research Center, Baqiyatallah University of Medical Sciences, Tehran, Iran; d Department of Nanotechnology, Agricultural Biotechnology Research Institute of Iran (ABRII), Agricultural Research, Education and Extension Organization (AREEO), Karaj, Iran

**Keywords:** Mesenchymal stem cells, Exosomes, SARS-CoV-2, COVID-19, Acute respiratory distress syndrome, Nanotechnology

## Abstract

The severe respiratory effects of the coronavirus disease 2019 (COVID-19) pandemic have necessitated the immediate development of novel treatments. The majority of COVID-19-related fatalities are due to acute respiratory distress syndrome (ARDS). Consequently, this virus causes massive and aberrant inflammatory conditions, which must be promptly managed. Severe respiratory disorders, notably ARDS and acute lung injury (ALI), may be treated safely and effectively using cell-based treatments, mostly employing mesenchymal stem cells (MSCs). Since the high potential of these cells was identified, a great deal of research has been conducted on their use in regenerative medicine and complementary medicine. Multiple investigations have demonstrated that MSCs and their products, especially exosomes, inhibit inflammation. Exosomes serve a critical function in intercellular communication by transporting molecular cargo from donor cells to receiver cells. MSCs and their derived exosomes (MSCs/MSC-exosomes) may improve lung permeability, microbial and alveolar fluid clearance, and epithelial and endothelial repair, according to recent studies. This review focuses on COVID-19-related ARDS clinical studies involving MSCs/MSC-exosomes. We also investigated the utilization of Nano-delivery strategies for MSCs/MSC-exosomes and anti-inflammatory agents to enhance COVID-19 treatment.

## Highlights

• MSCs/MSC-exosomse's anti-inflammatory, immune system-regulating, and tissue regeneration properties may make them effective COVID-19 treatments.
• Antiviral and inflammatory drugs can be delivered using MSC-exosome nanocarriers.
• The coalescence of nanoparticles with MSCs/MSC-exosomes may lead to a new COVID-19 treatment.

## 1 Background

Since December 2019, we have been witnessing the global outbreak of coronavirus 2019 (COVID-19) around the world. On March 11, 2020, the world health organization (WHO) declared COVID-19 a pandemic. COVID-19, the virus responsible for severe acute respiratory syndrome coronavirus 2 (SARS-CoV-2), is an enveloped, positive-sense, single-stranded RNA virus within the *Coronaviridae* family [[1], [2], [3]]. There is no doubt that COVID-19 is more contagious than either severe acute respiratory syndrome (SARS) or middle east respiratory syndrome (MERS), and it has a confusing clinical manifestation ranging from asymptomatic individuals to those with acute respiratory distress syndrome (ARDS) and pulmonary fibrosis, among other diseases, which has had disastrous implications for public health [4]. Age and underlying conditions such as diabetes, respiratory disease, hypertension disease, and cardiovascular disease are believed to increase the death risk in patients infected by the virus [[5], [6], [7]].

There are currently notices of an increase in the worldwide prevalence of the new COVID-19 variant (EG.5). Mutations in the EG.5 variant, known as Eris, increase the virus's ability to escape the immune system. The WHO first reported Eris on February 17, 2023. This is likely the cause of the recent rise in hospitalizations in Japan, New Zealand, South Korea, the United Kingdom, and the United States. Undoubtedly, a new variant of the coronavirus is spreading. With the onset of autumn and winter, it is likely that new mutations will reach the northern hemisphere. These new mutations could potentially lead to an increase in the spread and severity of respiratory illnesses. As a result, public health officials are closely monitoring the situation and preparing for potential outbreaks. These preparations include increasing testing capacity, ensuring sufficient vaccine supply, and updating public health guidelines [8,9].

Despite the great efforts worldwide to control COVID-19, vaccine research has clearly been important in reducing infection rates [[10], [11], [12]]. Since the beginning of the pandemic, a variety of treatment protocols have been used to treat patients around the world. However, the search for an effective COVID-19 treatment has not been very successful. From the beginning of the fight against this disease, drugs such as chloroquine/hydroxychloroquine, lopinavir/ritonavir, darunavir, ribavirin, remdesivir, favipiravir, interferon β1, convalescent plasma, molnupiravir, and paxlovid entered clinical trials, although some of these drugs are under investigation or even though their effectiveness has been ruled out. But the safety and potential effectiveness of many of them are still unknown [[13], [14], [15], [16], [17]]. One of the main reasons for the ineffectiveness of these treatments is the storm of cytokines in the lungs produced by the virus [18]. As the number of patients is on the rise and substantial mortality rate, novel therapeutic strategies are needed to reduce mortality and there is an improve recovery.

One of the possible ways of treatment is stem cell-based reconstructive medicine, which may be a suitable option for COVID-19 patients. As one of the most promising therapeutic approaches, stem cell therapy provides opportunities to treat diseases that were previously considered incurable [19]. Mesenchymal stem cells (MSCs) and mesenchymal stromal cells, also known as mesenchymal stromal stem cells, are frequently used interchangeably in the literature. However, it is important to recognize that there is a distinction between these two terms [20]. In 2005, the International Society for Cell & Gene Therapy (ISCT®) emphasized in a statement that the term “Mesenchymal Stem Cell” is not synonymous with or interchangeable with “Mesenchymal Stromal Cell” [21]. MSCs with two important features of self-renewal [22], differentiation and a function similar to progenitor cells can be proven [20]; on the other hand, mesenchymal stromal cells are a cell population with significant secretory ability and immunomodulatory and homing abilities [[23], [24], [25]]. This article defines MSCs and specifies the origin tissue for each MSC, as per ISCT® recommendation. MSCs derived from bone marrow and adipose tissue, which are abbreviated as BM-MSCs and AD-MSCs, respectively, are under discussion. Among cell therapies, MSCs are superior to other therapeutic strategies because they have a high rate of proliferation and allow invasive nature, as well as very few ethical and social problems [26,27]. The use of MSC therapy is widely used in the treatment of diabetes type 2, autoimmune diseases, spinal cord injuries, graft-versus-host disease (GVHD), as well as several other diseases [[28], [29], [30]]. They have the advantage of being accessible and isolable from a wide range of tissues, including bone marrow and adipose tissues such as the umbilical cord, dental pulp, menstrual blood, buccal fat pad, fetal liver, and so on [26,31]. Further, derivatives of MSCs such as microvesicles, exosomes, etc. Are also used today to induce cellular messages and therapeutic processes [[32], [33]]. The aim of the present review was to investigate the possible role of MSCs and their derived exosomes (MSCs/MSC-exosomes) in COVID-19-related ARDS. Moreover, we discussed the application of Nano-delivery strategies for MSCs/MSC-exosomes and anti-inflammatory agents in the management of COVID-19.

## 2 Therapeutic effects of MSCs on severe respiratory disorders

**Fig. 1:**
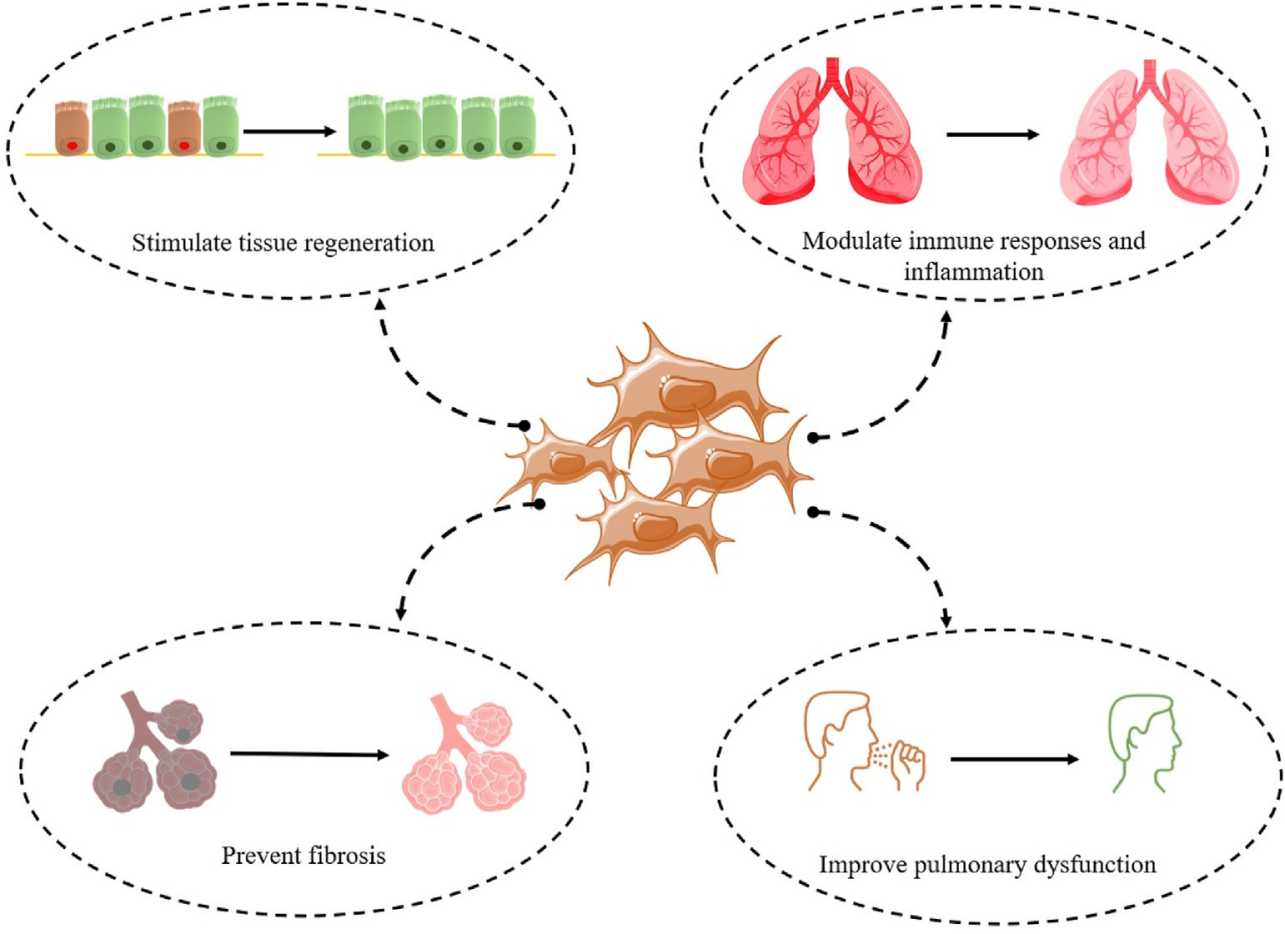
A schematic depiction of the function of MSCs in the treatment of severe respiratory disorders.

The therapeutic potential of MSCs for treating severe respiratory disorders has been extensively studied in pre-clinical trials, notably in ARDS and acute lung injury (ALI) animal models. MSCs modulate immune responses and inflammation, stimulate tissue regeneration, prevent fibrosis, and improve pulmonary dysfunction (Fig. 1) [[34], [35], [36], [37], [38]]. Intravenously injected MSCs will cause a portion of the population to get entrapped in the lungs. MSCs, when imprisoned in the lungs, produce a range of soluble mediators, such as antimicrobial peptides, anti-inflammatory cytokines, extracellular vesicles, and angiogenic growth factors [[39], [40]]. Furthermore, chemotaxis enables them to target injured lung tissues, allowing further exertion of their therapeutic effects [[41], [42]]. Studies show the infusion of MSCs reduces ALI caused by influenza A virus (H9N2) and increases mouse viability, mostly through attenuating host inflammation. MSCs were capable of modulating the levels of chemokines such as granulocyte-macrophage colony-stimulating factor (GMCSF), monocyte chemoattractant protein 1 (MCP-1), C-X-C motif chemokine ligand 1 (CXCL1), macrophage inflammatory protein-1 alpha (MIP-1-α), and cytokines including interleukin-1 alpha (IL-1α), IL-6, tumor necrosis factor alpha (TNF-α), and interferon gamma (IFN-γ) [43]. Busto et al. found that MSCs pre-activated with the blood of ALI patients generated high amounts of IL-10 and IL-1 receptor antagonist (IL-1RA), which decreased lung edema, inflammatory cell infiltration, and cytokine production [44]. MSCs protect the lungs from bleomycin-induced damage (a model of ALI) by producing IL-1RA, which reduces macrophage TNF release and prevents T-cell proliferation [45]. TNF-induced protein 6 helps MSCs minimize ALI [46].

## 3 Therapeutic effects of MSCs on COVID-19-related ARDS

There have been multiple infectious and nonpathogenic agents that can cause ARDS [47]. Among COVID-19 cases, the lungs appear to be the most injured organs [48]. Because the SARS-CoV-2 binding angiotensin converting enzyme 2 (ACE2) receptor is abundantly expressed on the surface of lung alveolar type II and capillary endothelial cells [49]. In the lungs, SARS-CoV-2 may induce a cytokine storm characterized by elevated levels of pro-inflammatory cytokines including IL-1β, IL-1RA, IL-2, IL-6, IL-7, granulocyte colony-stimulating factor (GCSF), IFN-γ, and TNF, as well as neutrophils and macrophages infiltration into the alveolar space [1,50]. This prolonged inflammatory response increases the formation of reactive oxygen species (ROS), which severely affect lung tissue and cause ARDS [51].

**Fig. 2:**
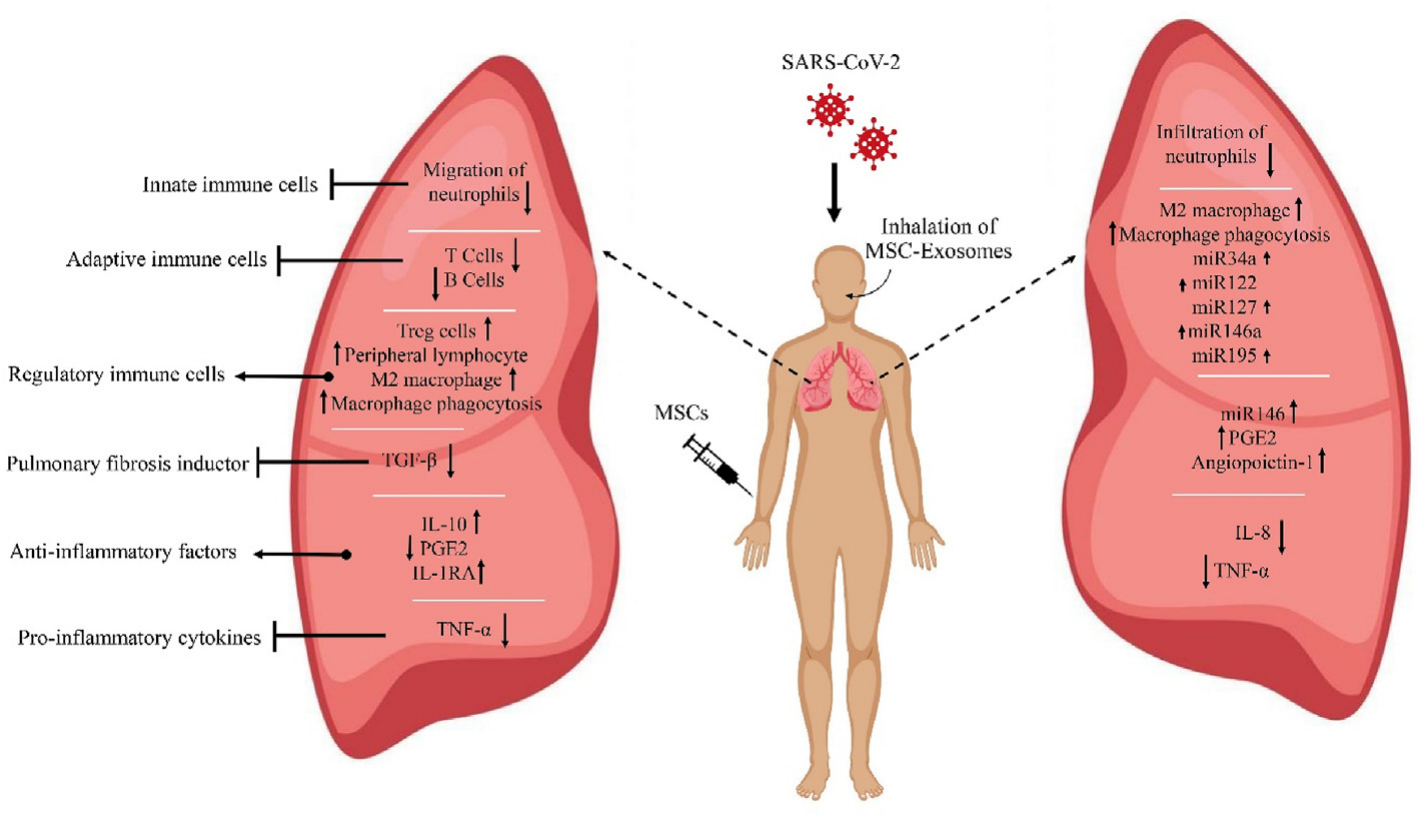
Mechanisms of lung injury in COVID-19 and potential therapeutic effects of MSCs/MSC-exosomes in COVID-19-related ARDS patients.

Due to their different mechanisms, MSCs have been shown in animal models of ARDS to inhibit inflammation and diminish lung damage. These cells influence macrophages by producing prostaglandin E2 (PGE2), which reduces the synthesis of inflammatory cytokines and elevates the production of IL-10 (Fig. 2). It therefore decreases the migration of neutrophils to the lungs and also significantly inhibit the generation of pro-inflammatory cytokines [[52], [53], [54], [55]]. By using their immunomodulatory properties, MSCs can inhibit cytokine storms, which are coordinated by cell-to-cell interactions and soluble factors released by them [56]. Through an assortment of immunomodulatory factors (e.g., transforming growth factor beta (TGF-β), PGE2, and soluble human leukocyte antigen-G molecules (HLA-G5)), MSCs can inhibit the activation of T cells [[57], [58]]. As compared to monoclonal antibodies, MSCs can affect multiple cytokines at once. Research has shown that MSCs reduce inflammatory responses and defend the host against cytokine storms with lowered mortality without causing serious side effects [[59], [60], [61]]. MSCs are also capable of increasing regulatory T cells and altering the M1-M2 phenotype of macrophages [62]. MSCs may avoid lung cell death and regenerate them by generating some of the growth factors. After surviving the acute phase of the disease, ARDS patients develop lung fibrosis; therefore, this trait is significant [[63], [64]]. MSCs may recover the protein accessibility of epithelial cells by secreting angiopoietin-1. By transferring the microvesicles, these cells may improve macrophages phagocytosis ability [62].

MSCs have recently been identified as a prospective method for reducing immune overactivation in COVID-19 patients [65]. COVID-19 causes an exaggerated immune response in the body by producing large quantities of various inflammatory factors, including several cytokines, chemokines, and immunoreactive molecules. MSC therapy might be able to prevent the development of a cytokine storm triggered by an activated immune system. The reparative properties of stem cells may be beneficial for endogenous repair [66].

With the assistance of MSCs, the pulmonary microenvironment might be restored, safeguarding the alveolar epithelial cells. Consequently, pulmonary fibrosis of the lungs might be averted, which could result in a treatment for COVID-19-caused pneumonia [18]. After MSC infusion, peripheral lymphocytes rose and C-reactive protein (CRP) levels fell in COVID-19 patients. Overactivated cytokine-secreting immune cells were reduced in 6 days. Regulatory dendritic cells (CD14+CD11bmid) increased following MSC therapy. TNF-α levels decreased in MSC-treated COVID-19 patients, whereas IL-10 levels rose compared to conventionally treated patients. The data indicated that pulmonary compliance had recovered. Interestingly, MSCs are resistant to SARS-CoV-2 infection because they lack ACE-2 and transmembrane serine protease 2 (TMPRSS-2) receptors [56].

In 2020, Leng et al. conducted the first investigation on stem cell therapy for COVID-19; this study examined seven COVID-19 patients. The results of this study showed that it promoted rehabilitation after giving intravenous clinical-grade MSCs. Each patient received 1 × 10^6^ MSCs per kg of body weight by intravenous infusion. After 2–4 days of MSC injection, all early symptoms such as high fever, weakness, shortness of breath, and hypoxia disappeared, and lung function improved [56]. Alturi et al. reported the treatment of a COVID-19 patient with human umbilical cord (hUC)-MSCs. This patient was a 65-year-old from China. The treatment protocol was that 3 doses of 5 × 10^7^ million each were injected 3 days apart. No adverse or hypersensitive reactions were reported after treatment [67]. In addition, a phase 1 clinical trial of hUC-MSCs showed that treatment of COVID-19 with three intravenous infusions of hUC-MSCs (3 × 10^7^ cells per infusion) could be effective in reducing symptoms [68]. Registered clinical trials to date have used a variety of stem cell sources. Nearly half (40%) of the studies did not cite the source of MSCs, but the most common source of MSCs was umbilical cord MSCs (26%). The least used sources are cord blood and placenta (2%). 43 MSC-based clinical trials are currently registered with a positive outcome for curing SARS-CoV-2 patients [69]. Approximately 20 clinical trials have been registered on clinicaltrials.gov to investigate the use of MSCs in COVID-19-related ARDS patients (Table 1).

**Table 1:** MSC therapy in COVID-19-induced ARDS patients (https://clinicaltrials.gov/).

Trail identification	Title	Clinical phase; Patient (n)	Source of MSC	Recruitment status
NCT04416139	Mesenchymal stem cell for ARDS due for COVID-19	2; 10	Umbilical cord-MSCs	Unknown
NCT04565665	Cord blood-derived MSCs for the treatment of COVID-19 related ARDS	1/2; 70	Umbilical cord-MSCs	Recruiting
NCT04456361	Use of MSCs in ARDS caused by COVID-19	1; 9	Wharton's jelly-MSCs	Active, not recruiting
NCT04625738	Efficacy of infusions of MSC from Wharton jelly in the SARS-CoV-2 (COVID-19) related ARDS	2; 30	Wharton's jelly-MSCs	Completed
NCT04629105	Regenerative medicine for COVID-19 and flu-elicited ARDS using longeveron MSCs (LMSCs) (recover)	1; 70	Longeveron MSCs	Active, not recruiting
NCT04390152	Safety and efficacy of intravenous Wharton's jelly derived MSCs in ARDS due to COVID 19	1/2; 40	Wharton's jelly-MSCs	Unknown
NCT04494386	UC lining stem cells (ULSC) in patients with COVID-19 ARDS	1/2; 17	Umbilical cord-MSCs	Active, not recruiting
NCT04452097	Use of hUC-MSC product (BX-U001) for the treatment of COVID-19 with ARDS	1/2; 39	Human umbilical Cord-MSCs	Not yet recruiting
NCT04377334	MSCs in inflammation-resolution programs of COVID-19 induced ARDS	2; 40	Bone marrow-MSCs	Not yet recruiting
NCT03042143	Repair of ARDS by stromal cell administration (realist) (COVID-19)	1/2; 129	Human umbilical cord derived CD362 enriched MSCs	Recruiting
NCT04371393	MSCs in COVID-19 ARDS	3; 223	Remestemcel-L	Terminated
NCT04333368	Cell therapy using umbilical cord-derived MSCs in SARS-CoV-2-related ARDS	1/2; 47	Umbilical cord Wharton's jelly-derived human	Completed
NCT04447833	Mesenchymal stromal cell therapy for the treatment of ARDS	1; 7	MSCs-KI-MSC-PL-205	Active, not recruiting
NCT04466098	Multiple dosing of mesenchymal stromal cells in patients with ARDS (COVID-19)	2; 9	MSCs	Active, not recruiting
NCT04400032	Cellular immuno-therapy for COVID-19 ARDS (CIRCA-19)	1/2; 15	MSCs	Completed
NCT04615429	Clinical trial to assess the efficacy of MSC in patients with ARDS due to COVID-19	2; 20	MSCs	Recruiting
NCT04525378	MSC-based therapy in COVID-19-associated ARDS	1; 20	MSCs	Unknown
NCT04399889	hCT-MSCs for COVID19 ARDS	1/2; 12	Human cord tissue-MSCs	Terminated
	MultiStem administration for COVID-19 induced ARDS (MACoVIA)	2/3; 400	MultiStem	Recruiting
NCT04355728	Use of UC-MSCs for COVID-19 patients	1/2; 24	Umbilical cord-MSCs	Completed

## 4 MSC-exosomes-based therapies for COVID-19-related ARDS

Exosomes are a subset of extracellular vesicles (EVs) that shuttle molecular cargoes from donor cells to recipient cells and play a crucial role in mediating intercellular communication [70]. The delivery of various biological molecules like microRNA (miRNA) and proteins into target cells is a crucial function of exosomes in physiological and pathological conditions. This issue plays a critical role in cell-to-cell communications [71]. Unlike apoptotic bodies and microvesicles, which are produced from the surface of the cell, exosomes are produced by endosomal pathways and contain the cytoplasmic content of the original cell. Thus, they mimic all the properties of the cells they are descended from Ref. [72].

Exosomes are important paracrine effectors secreted by MSCs. Because their biological cargo is similar to that of their progenitor cells and they can keep their regenerative properties, they have been looked at as a possible replacement for MSCs in the treatment of different diseases [73]. Exosomes are nontoxic, low-immunogenic, stable, easy to store, and can be manufactured as an off-the-shelf product. These benefits have led to special attention being paid to these cellular products [74]. Exosomes move bioactive membrane and cytoplasmic parts from the cell that made them to the cell that receives them. This happens when the plasma membranes of the two cells fuse. MSC-exosomes, in addition to the unique properties mentioned, also have the property of modulating the immune system. Therefore, these exosomes can be introduced as an interesting tool for therapeutic intervention to deal with the current situation of COVID-19 [[75], [76]].

Investigations have shown that the MSC-exosomes were able to reprogram macrophages in the inflammatory environment of ARDS toward reducing the production of TNF-α and IL-8 as the major pro-inflammatory cytokines (Fig. 2). M1-M2 polarization of macrophages and also improving and enhancing their phagocytosis effects and oxidative phosphorylation [[77], [78], [79]]. Several miRNAs, like miR-34a, miR-122, miR-124, miR-127, miR-146a, and miR-195, are found in MSC-exosomes. These miRNAs are thought to be responsible for the immune-modulating effects of MSCs [[80], [81]]. Researchers found that an anti-inflammatory miRNA, miR-146, packaged into MSC-exosomes and induced macrophages to become M2 phenotype cells [82]. Studies have shown that MSC-exosomes can induce the production of PGE2. PGE2 is considered a critical key for changing the M1 phenotype to the M2 phenotype of macrophages [82]. MSC-exosomes in the environmental oxidative stress in animal models of chronic obstructive pulmonary disease (COPD) modulated the function of macrophages through mitochondrial transfer and blocked [78]. Several in vitro and in vivo studies [[83], [84], [85]] have shown that MSC-exosomes can suppress the immune system and repair damaged tissues in a number of lung diseases. Studies have shown that MSC-exosomes, due to high levels of angiopoietin-1 mRNA, which is an anti-inflammatory and anti-permeability agent, can reduce the permeability of pulmonary edema and the infiltration of neutrophils [[76], [86]]. The sodium channel and keratinocyte growth factor (KGF) are upregulated in alveolar epithelial cells after MSC-exosome administration, increasing alveolar fluid clearance (AFC) [76]. Furthermore, in vivo studies revealed that MSC-exosomes could reduce pulmonary hypoxic hypertension [[87], [88], [89]].

Based on the results of these studies, MSC-exosomes may increase recovery in COVID-19-related ARDS patients by rebalancing immune system dysfunction and cytokine storm management (Table 2).

**Table 2:** COVID-19-induced ARDS patients treated with MSC-exosomes (https://clinicaltrials.gov/).

Trail identification	Title	Clinical phase; Patient (n)	Source of exosome	Recruitment status
NCT04276987	A pilot clinical study on inhalation of MSCs exosomes treating severe novel coronavirus pneumonia	1; 24	MSC-derived exosomes	Completed
NCT04491240	Evaluation of safety and efficiency of method of exosome inhalation in SARS-CoV-2 associated pneumonia (COVID-19EXO)	1/2; 30	MSC-derived exosomes	Completed
NCT04602442	Safety and efficiency of method of exosome inhalation in COVID-19 associated pneumonia (COVID-19EXO2)	2; 90	MSC-derived exosomes	Unknown
NCT04798716	The use of exosomes for the treatment of ARDS or novel coronavirus pneumonia caused by COVID-19 (ARDOXSO)	1/2; 55	MSC-derived exosomes	Not yet recruiting
NCT05216562	Efficacy and safety of Exosome-MSC therapy to reduce hyper-inflammation in moderate COVID-19 patients (EXOMSC-COV19)	2/3; 60	MSC-derived exosomes	Recruiting

## 5 Antiviral properties of MSCs/MSC-exosomes

According to several studies, MSCs may have antiviral properties. It seems that MSCs can limit viral replication by activating the stimulator of the interferon gene/TANK-binding kinase 1 (STING/TBK1) signaling pathway. This signaling, which leads to IFN-γ production, may be responsible for the antiviral response of MSCs [90]. In contrast, several studies have shown that indoleamine 2,3-dioxygenase (IDO) released by MSCs may contribute to their antiviral activity. Activated by inflammatory cytokines, IDO-positive MSCs exhibited antibacterial mediator activity versus pathogens such as cytomegalovirus (CMV) and herpes simplex virus type 1 (HSV-1) [[91], [92], [93]]. MSCs express a variety of IFN-stimulated genes (ISGs), such as the interferon-induced transmembrane family (IFITM), spermidine/spermine N1-acetyltransferase 1 (SAT1), interferon-alpha inducible protein 6 (IFI6), phorbol-12-myristate-13-acetate-induced protein 1 (PMAIP1), ISG15, C–C motif chemokine ligand 2 (CCL2), p21/cyclin dependent kinase inhibitor 1a (CDKN1a), etc., many of which are recognized for their antiviral properties. Interferon-induced transmembrane family proteins are unusual in that they prevent infection before the virus can enter the lipid bilayer of the cell. This effect attributed to IFITM proteins inhibited the infection of cultured cells by Ebola, influenza A, dengue, and SARS [94]. Antimicrobial impact is an additional significant function of MSC in the treatment of ARDS. In particular, MSC may augment the phagocytic activity of monocytes, neutrophils, and macrophages [[95], [96]]. This intervention may be necessary to prevent or treat opportunistic bacterial infections in instances of virally-caused damage, such as COVID-19.

It was shown in one study that MSC-exosomes might increase virus clearance by CD8^+^ T cells in neonatal mice infected with influenza A virus [97]. The antiviral activity of MSC-exosomes in the pig model of influenza virus has also been reported. These exosomes reduce the inflammatory status by inhibiting the expression of cytokines and chemokines in pig models [85]. Exosomes, for example, can be used to deliver antiviral drugs. Engineered exosomes containing antibodies or nanoparticles of anti-COVID-19 can also be designed. It can be said as a scenario, when ACE2 is present on the surface of lung-derived exosomes, viral particles are trapped before infection [[98], [99], [100]].

## 6 Anti-fibrosis properties of MSCs/MSC-exosomes and therapeutic effect on long COVID-19

In the advanced phases of SARS-CoV-2 infection, there is a disturbance in the integrity of the barrier between lung epithelial and endothelial cells. This disruption results in the thickening of alveolar walls and the development of fibrosis. These changes occur due to the infiltration of monocytes and alveolar macrophages, which subsequently impairs the exchange of gases between the alveoli and capillaries, leading to a state of hypoxia [[101], [102]]. MSCs/MSC-exosomes have emerged as a prominent therapeutic strategy for the treatment of pulmonary fibrosis due to their ease of isolation, little immunogenicity, reparative attributes, and anti-inflammatory characteristics [74]. MSCs may prevent lung fibrosis by migrating to the damaged site and releasing paracrine substances. Exosomes, in this context, also fulfill significant autocrine and paracrine functions in intercellular communication by transmitting biological information to neighboring cells [[73], [103]]. MSC-exosomes regulate epithelial and endothelial cell permeability. Additionally, these substances reduce inflammation and aid tissue healing. Previous studies have shown that MSC-exosomes have the ability to impede pro-inflammatory processes. Moreover, these exosomes have been linked to the mitigation of oxidative stress, pulmonary fibrosis, and the remodeling characteristic of inflammatory lung diseases [104]. The findings of a meta-analysis study have shown that the transplantation of MSCs led to a significant reduction in lung fibrosis levels in comparison to the control group [105]. In a recent study focused on pulmonary fibrosis, researchers investigated the efficacy of delivering exosomes by inhalation as a means of promoting lung regeneration. The findings of this study demonstrated encouraging outcomes [106]. Notably, the first AETHER clinical trial demonstrated the safety of human allogeneic bone marrow in MSCs following an IV injection into patients with idiopathic pulmonary fibrosis, paving the way for MSCs to treat respiratory illnesses. Nine patients diagnosed with mild to moderate idiopathic pulmonary fibrosis participated in the AETHER study, which was a phase I, single-center, non-randomized, and non-placebo-controlled trial [107]. It is anticipated that MSCs/MSC-exosomse will be successful in the treatment of COVID-19 infection in the near future due to their unique properties, such as anti-inflammatory capacity, immune system regulator capacity, and tissue regeneration capacity.

## 7 Nano-based delivery systems for MSCs/MSC-exosomes in COVID-19

**Fig. 3:**
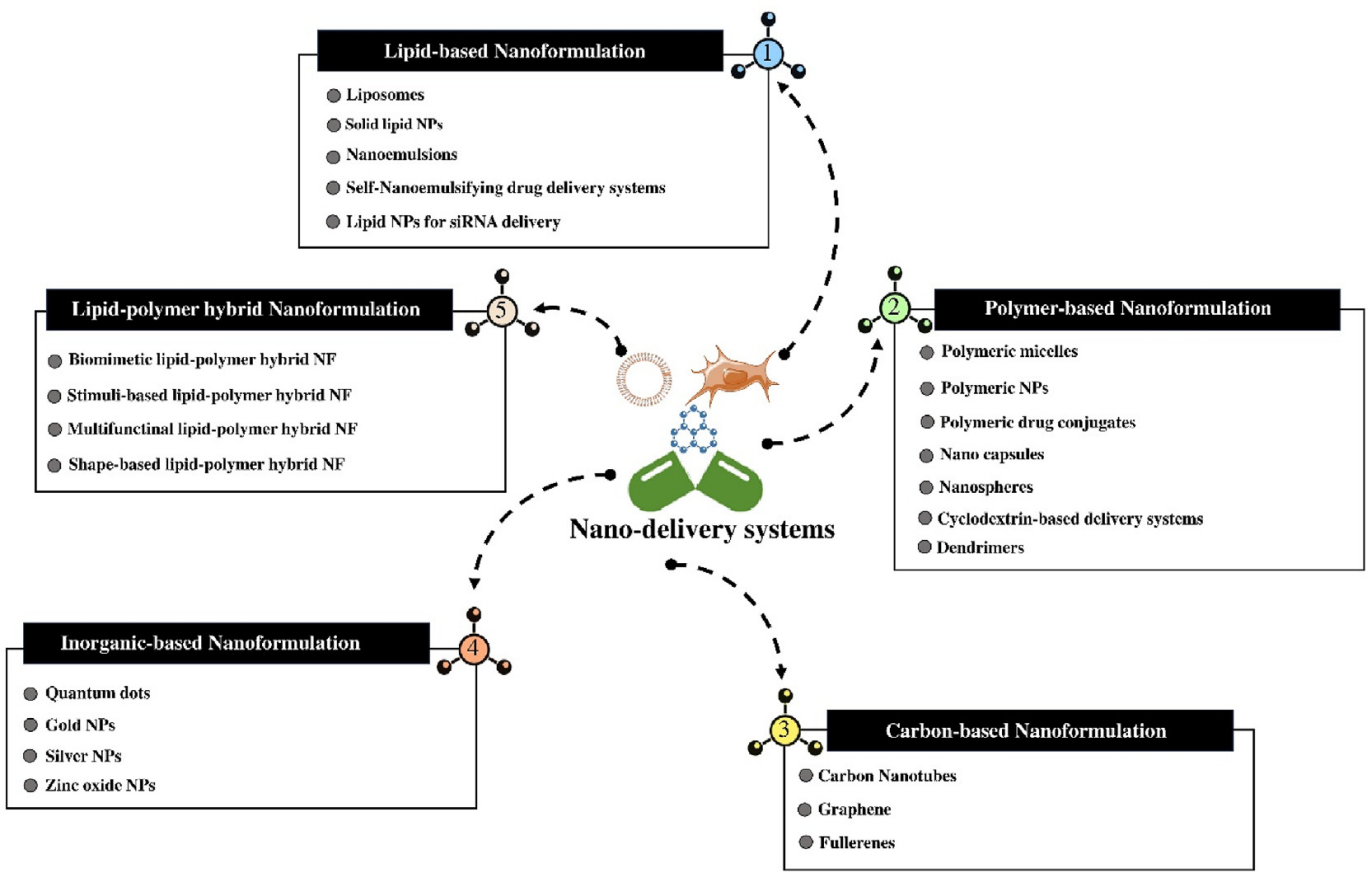
The five types of Nano-delivery systems are shown schematically: 1. Lipid-based Nano-formulation; 2. Polymer-based Nano-formulation; 3. Carbon-based Nano-formulation; 4. Inorganic-based Nano-formulation; 5. Lipid-polymer hybrid Nano-formulation. NF, Nano-formulation; NPs, Nanoparticles.

Due to their sustained release, selectivity, and specificity, nanocarriers are a versatile platform for the delivery of pharmaceuticals, vaccines, and cells. MSCs/MSC-exosomes can be delivered more efficiently and effectively using nanotechnology to treat COVID-19 and reduce mortality (Fig. 3). As previously indicated, the cytokine storm poses a significant threat to COVID-19 patients, and it must be contained immediately. According to a study, Nano-synthetic stem cells (LIFNano) may mitigate the COVID-19-induced cytokine storm. LIFNano, a new cell-based treatment option, rejuvenates damaged tissues and suppresses cytokine storms in pneumonia in large volumes and off-the-shelf [108]. MSC-exosomes are nanosized in comparison to MSCs. This enables them to cross the blood-brain barrier, thereby preventing the occurrence of pulmonary embolism following transplantation of MSCs [109]. Interestingly, MSC-exosomes can be used as nanocarriers to deliver antiviral drugs and anti-inflammatory agents [110]. Hence, they are regarded as a nanoscale delivery platform for microRNAs, which by nature may target and modify genes associated with the cytokine storm, a severe immune response found in COVID-19 patients [111].

The use of Nano-formulations has the potential to augment the specific delivery and therapeutic effectiveness of anti-inflammatory and antiviral agents [112]. The Nano-delivery systems may be classified into five distinct categories: 1. Nano-formulations based on lipids; 2. Nano-formulations based on polymers; 3. Nano-formulations based on carbons; 4. Nano-formulations based on inorganic compounds; 5. Lipid-polymer hybrid Nano-formulations (Fig. 3) [113]. Multiple research studies demonstrate that Nano-formulation of anti-inflammatory compounds increases their effectiveness versus COVID-19 [[114], [115], [116]]. For instance, chitosan, as a Nano-formulation based on polymers [117], serves an important function in the delivery of drugs to pulmonary tissue in infectious diseases. Chitosan transports medications in the lungs because of its mucoadhesiveness, penetration, and site-specificity [118]. Consequently, adding chitosan to MSCs/MSC-exosomes might improve their therapeutic effectiveness, penetration, adhesion, and targeting. Hence, the coalescence of these nanoparticles with MSCs/MSC-exosomes has promise for establishing a novel therapeutic approach for COVID-19 [119]. The researchers conducted a study whereby they utilized Nano-formulation to improve the stability and solubility of curcumin and to examine its impact on inflammatory cytokines in COVID-19 patients. Their results showed that patients exposed to Nano-curcumin exhibited a significant reduction in the mRNA expression of IL-6 and IL-1β [120]. Meng et al. proposed a Nano-formulation of dexamethasone (Nano-dexamethasone) to enhance the alleviation of COVID-19 disorders in animal models. In a mouse model of acute pneumonia, Nano-dexamethasone augmented the anti-inflammatory activity of dexamethasone. In addition, a 10-fold lower dose of inhaled Nano-dexamethasone had even greater effects on pulmonary inflammation and injury in rhesus macaques infected with SARS-CoV-2 than intravenous dexamethasone [121].

## 8 Challenges and hopes

Stem cell therapy always faces challenges. MSCs can be isolated and cultured from most tissues of the human body, but there are limitations in clinical terms and the use of these cells in clinical trials. For example, the limited availability of the appropriate source and the invasiveness of some methods of obtaining cells have made it difficult to access the appropriate amount and quality of these cells. Although MSCs isolated from different tissues have many common features, their biological functions may be slightly different. Therefore, special attention should be paid to the proper access and correct processing of these cells [[122], [123]].

One of the most important challenges of using mesenchymal cells is their fate in the patient's body. After intravenous injection, cells may go to other tissues with blood circulation, and there is a possibility that these cells will receive different differentiation signals. Another challenge is that when MSCs are injected systemically, we quickly see the presence of these cells in the blood circulation, which may cause embolism. Another ambiguity regarding the proper use of MSCs is the possibility of clearing these cells by the immune system and macrophages in the blood circulation. Another concern when using MSCs is cell heterogeneity. Different origins of MSCs can lead to variable results [[123], [124], [125]].

In order to correct the path of the presence of stem cells in the specific place and as desired by the researchers, several strategies have been used to improve the homing ability of MSCs, for example, targeted drug delivery, magnetic guidance, gene modification, laboratory priming, surface modification cells, and radiotherapy techniques [[126], [127]]. In addition to all these cases, it may take several days or weeks to prepare MSCs in appropriate laboratory conditions until reaching the desired therapeutic dose, and when there are urgent conditions, managing time, cost, GMP-grade reagents, and proper quality testing is another important challenge that must be taken into account [[128], [129]].

Given the lack of toxicity of exosomes to cells, it is highly advantageous to use these vectors, whether they are extracted directly from MSCs or engineered. One of the upcoming challenges in the use of exosomes is the technology of exosome production and their quality control. Although it was reported that the GMP production of exosomes is possible on a small scale, their standard preparation has limitations on a large scale. It should be noted that currently, there is no agreement on technical standards for exosome production and isolation in the world [[130], [131], [132], [133]].

Taking into account all relevant factors, there is a chance that MSC-based cell therapy and its derivatives, such as exosomes, will be expanded in the near future to treat COVID-19-induced ARDS.

## 9 Conclusion

Considering that there is currently no effective treatment for serious patients in critical conditions, MSCs can be a suitable candidate for modulating the immune and anti-inflammatory responses of these patients. The inherent ability of these cells to repair damaged tissue, especially the anti-fibrosis and angiogenic properties of these cells and their special secretions such as exosomes, can be very promising for the repair of lung damage. Clinical studies have shown that MSCs and their derivatives, such as exosomes and microvesicles, can be effective in the treatment of COVID-19-related ARDS patients (especially critically ill patients) in future treatment methods. Suppose the challenges of treating patients with COVID-19 with stem cells are addressed. In that case, we will have a promising opportunity to improve inflammation, prevent long-term lung disability, contribute to lung tissue recovery, and reduce mortality. So, it seems that more research in this area can be precious. Undoubtedly, by combining MSC-based therapies with other therapies, effective strategies to combat COVID-19 can be developed in the future.

## Author contribution statement

Mohsen Ghiasi: conceptualization, writing-original draft preparation, data curation. Peyman Kheirandish Zarandi: writing-original draft preparation, data curation. Abdolreza Dayani: writing-original draft preparation and data curation. Ali Salimi: writing-reviewing and editing. Ehsan Shokri: project administration, supervision, conceptualization, writing-reviewing and editing. All authors contributed to the paper and approved the submitted version.

## Funding

This research did not receive any specific grant from funding agencies in the public, commercial, or not-for-profit sectors.

## Data availability statement

Not applicable.

## Declaration of competing interest

The authors declare that they have no known competing financial interests or personal relationships that could have appeared to influence the work reported in this paper.

## References

[1] Huang C., Wang Y., Li X., Ren L., Zhao J., Hu Y. (2020). Clinical features of patients infected with 2019 novel coronavirus in Wuhan, China. Lancet.

[2] Guan W-j, Ni Z-y, Hu Y., Liang W-h, Ou C-q, He J-x (2020). Clinical characteristics of coronavirus disease 2019 in China. N Engl J Med.

[3] Zhou P., Yang X.-L., Wang X.-G., Hu B., Zhang L., Zhang W. (2020). A pneumonia outbreak associated with a new coronavirus of probable bat origin. Nature.

[4] Li Z., Niu S., Guo B., Gao T., Wang L., Wang Y. (2020). Stem cell therapy for COVID-19, ARDS and pulmonary fibrosis. Cell Prolif.

[5] Aggarwal G., Cheruiyot I., Aggarwal S., Wong J., Lippi G., Lavie C.J. (2020). Association of cardiovascular disease with coronavirus disease 2019 (COVID-19) severity: a meta-analysis. Curr Probl Cardiol.

[6] Iaccarino G., Grassi G., Borghi C., Ferri C., Salvetti M., Volpe M. (2020). Age and multimorbidity predict death among COVID-19 patients: results of the SARS-RAS study of the Italian Society of Hypertension. Hypertension.

[7] Zinatizadeh M.R., Zarandi P.K., Ghiasi M., Kooshki H., Mohammadi M., Amani J. (2023). Immunosenescence and inflamm-ageing in COVID-19. Ageing Res Rev.

[8] Dyer O. (2023).

[9] Parums D.V. (2023). A rapid global increase in COVID-19 is due to the emergence of the EG. 5 (eris) subvariant of omicron SARS-CoV-2. Med Sci Mon Int Med J Exp Clin Res.

[10] Chung J.Y., Thone M.N., Kwon Y.J. (2021). COVID-19 vaccines: the status and perspectives in delivery points of view. Adv Drug Deliv Rev.

[11] Koff W.C., Schenkelberg T., Williams T., Baric R.S., McDermott A., Cameron C.M. (2021). Development and deployment of COVID-19 vaccines for those most vulnerable. Sci Transl Med.

[12] Hacisuleyman E., Hale C., Saito Y., Blachere N.E., Bergh M., Conlon E.G. (2021). Vaccine breakthrough infections with SARS-CoV-2 variants. N Engl J Med.

[13] Li H., Wang Y., Xu J., Cao B. (2020). Potential antiviral therapeutics for 2019 novel coronavirus. Zhonghua jie he he hu xi za zhi= Zhonghua jiehe he huxi zazhi= Chin J Tuberc Respir Dis.

[14] Shen C., Wang Z., Zhao F., Yang Y., Li J., Yuan J. (2020). Treatment of 5 critically ill patients with COVID-19 with convalescent plasma. JAMA.

[15] Cohen M.S. (2020). Hydroxychloroquine for the prevention of Covid-19—searching for evidence. N Engl J Med.

[16] Zarandi P.K., Zinatizadeh M.R., Zinatizadeh M., Yousefi M.H., Rezaei N. (2021). SARS-CoV-2: from the pathogenesis to potential anti-viral treatments. Biomed Pharmacother.

[17] Zinatizadeh M.R., Zarandi P.K., Zinatizadeh M., Yousefi M.H., Amani J., Rezaei N. (2022). Efficacy of mRNA, adenoviral vector, and perfusion protein COVID-19 vaccines. Biomed Pharmacother.

[18] Rajarshi K., Chatterjee A., Ray S. (2020). Combating COVID-19 with mesenchymal stem cell therapy. Biotechnol Rep.

[19] Golchin A., Farahany T.Z. (2019). Biological products: cellular therapy and FDA approved products. Stem Cell Rev Rep.

[20] Viswanathan S., Shi Y., Galipeau J., Krampera M., Leblanc K., Martin I. (2019). Mesenchymal stem versus stromal cells: international society for cell & gene therapy (ISCT®) mesenchymal stromal cell committee position statement on nomenclature. Cytotherapy.

[21] Horwitz E., Le Blanc K., Dominici M., Mueller I., Slaper-Cortenbach I., Marini F.C. (2005). Clarification of the nomenclature for MSC: the international society for cellular therapy position statement. Cytotherapy.

[22] Bianco P., Cao X., Frenette P.S., Mao J.J., Robey P.G., Simmons P.J. (2013). The meaning, the sense and the significance: translating the science of mesenchymal stem cells into medicine. Nat Med.

[23] Dennis J.E., Caplan A.I. (2004). Advances in mesenchymal stem cell biology. Curr Opin Orthop.

[24] Le Blanc K., Mougiakakos D. (2012). Multipotent mesenchymal stromal cells and the innate immune system. Nat Rev Immunol.

[25] Kallmeyer K., Pepper M.S. (2015). Homing properties of mesenchymal stromal cells. Expet Opin Biol Ther.

[26] Hade M.D., Suire C.N., Suo Z. (2021). Mesenchymal stem cell-derived exosomes: applications in regenerative medicine. Cells.

[27] Musiał-Wysocka A., Kot M., Majka M. (2019). The pros and cons of mesenchymal stem cell-based therapies. Cell Transplant.

[28] Golchin A., Farahany T.Z., Khojasteh A., Soleimanifar F., Ardeshirylajimi A., therapy (2019). The clinical trials of mesenchymal stem cell therapy in skin diseases: an update and concise review. Curr Stem Cell Res Ther.

[29] Novello S., Debouche A., Philippe M., Naudet F., Jeanne S. (2020). Clinical application of mesenchymal stem cells in periodontal regeneration: a systematic review and meta-analysis. J Periodontal Res.

[30] Zhao K., Liu Q. (2016). The clinical application of mesenchymal stromal cells in hematopoietic stem cell transplantation. J Hematol Oncol.

[31] Basiri A., Mansouri F., Azari A., Ranjbarvan P., Zarein F., Heidari A. (2021). Stem cell therapy potency in personalizing severe COVID-19 treatment. Stem Cell Rev Rep.

[32] Huldani H., Jasim S.A., Bokov D.O., Abdelbasset W.K., Shalaby M.N., Thangavelu L. (2022). Application of extracellular vesicles derived from mesenchymal stem cells as potential therapeutic tools in autoimmune and rheumatic diseases. Int Immunopharm.

[33] Liu H., Deng S., Han L., Ren Y., Gu J., He L. (2022). Mesenchymal stem cells, exosomes and exosome-mimics as smart drug carriers for targeted cancer therapy. Colloids Surf B Biointerfaces.

[34] Weiss A.R.R., Dahlke M.H. (2019). Immunomodulation by mesenchymal stem cells (MSCs): mechanisms of action of living, apoptotic, and dead MSCs. Front Immunol.

[35] Walter J., Ware L.B., Matthay M.A. (2014). Mesenchymal stem cells: mechanisms of potential therapeutic benefit in ARDS and sepsis. Lancet Respir Med.

[36] Curley G.F., Jerkic M., Dixon S., Hogan G., Masterson C., O'Toole D. (2017). Cryopreserved, xeno-free human umbilical cord mesenchymal stromal cells reduce lung injury severity and bacterial burden in rodent Escherichia coli–induced acute respiratory distress syndrome. Crit Care Med.

[37] Yang Y., Hu S., Xu X., Li J., Liu A., Han J. (2016). The vascular endothelial growth factors-expressing character of mesenchymal stem cells plays a positive role in treatment of acute lung injury in vivo. Mediat Inflamm.

[38] Goolaerts A., Pellan-Randrianarison N., Larghero J., Vanneaux V., Uzunhan Y., Gille T. (2014). Conditioned media from mesenchymal stromal cells restore sodium transport and preserve epithelial permeability in an in vitro model of acute alveolar injury. Am J Physiol Lung Cell Mol Physiol.

[39] Lee R.H., Pulin A.A., Seo M.J., Kota D.J., Ylostalo J., Larson B.L. (2009). Intravenous hMSCs improve myocardial infarction in mice because cells embolized in lung are activated to secrete the anti-inflammatory protein TSG-6. Cell Stem Cell.

[40] Hu S., Park J., Liu A., Lee J., Zhang X., Hao Q. (2018). Mesenchymal stem cell microvesicles restore protein permeability across primary cultures of injured human lung microvascular endothelial cells. Stem Cell Transl Med.

[41] Karp J.M., Teo G.S.L. (2009). Mesenchymal stem cell homing: the devil is in the details. Cell Stem Cell.

[42] Liesveld J.L., Sharma N., Aljitawi O.S. (2020). Stem cell homing: from physiology to therapeutics. Stem Cell.

[43] Li Y., Xu J., Shi W., Chen C., Shao Y., Zhu L. (2016). Mesenchymal stromal cell treatment prevents H9N2 avian influenza virus-induced acute lung injury in mice. Stem Cell Res Ther.

[44] Bustos M.L., Huleihel L., Meyer E.M., Donnenberg A.D., Donnenberg V.S., Sciurba J.D. (2013). Activation of human mesenchymal stem cells impacts their therapeutic abilities in lung injury by increasing interleukin (IL)-10 and IL-1RN levels. Stem Cell Transl Med.

[45] Ortiz L.A., DuTreil M., Fattman C., Pandey A.C., Torres G., Go K. (2007). Interleukin 1 receptor antagonist mediates the antiinflammatory and antifibrotic effect of mesenchymal stem cells during lung injury. Proc Natl Acad Sci USA.

[46] Danchuk S., Ylostalo J.H., Hossain F., Sorge R., Ramsey A., Bonvillain R.W. (2011). Human multipotent stromal cells attenuate lipopolysaccharide-induced acute lung injury in mice via secretion of tumor necrosis factor-α-induced protein 6. Stem Cell Res Ther.

[47] Taghavi-Farahabadi M., Mahmoudi M., Soudi S., Hashemi S.M. (2020). Hypothesis for the management and treatment of the COVID-19-induced acute respiratory distress syndrome and lung injury using mesenchymal stem cell-derived exosomes. Med Hypotheses.

[48] Barros I., Silva A., de Almeida L.P., Miranda C.O. (2021). Mesenchymal stromal cells to fight SARS-CoV-2: taking advantage of a pleiotropic therapy. Cytokine Growth Factor Rev.

[49] Jia H.P., Look D.C., Shi L., Hickey M., Pewe L., Netland J. (2005). ACE2 receptor expression and severe acute respiratory syndrome coronavirus infection depend on differentiation of human airway epithelia. J Virol.

[50] Liu J., Li S., Liu J., Liang B., Wang X., Wang H. (2020). Longitudinal characteristics of lymphocyte responses and cytokine profiles in the peripheral blood of SARS-CoV-2 infected patients. EBioMedicine.

[51] Ware L.B., Matthay M.A. (2000). The acute respiratory distress syndrome. N Engl J Med.

[52] Gupta N., Su X., Popov B., Lee J.W., Serikov V., Matthay M.A. (2007). Intrapulmonary delivery of bone marrow-derived mesenchymal stem cells improves survival and attenuates endotoxin-induced acute lung injury in mice. J Immunol.

[53] Devaney J., Horie S., Masterson C., Elliman S., Barry F., O’Brien T. (2015). Human mesenchymal stromal cells decrease the severity of acute lung injury induced by E. coli in the rat. Thorax.

[54] Lee J.W., Fang X., Gupta N., Serikov V., Matthay M.A. (2009). Allogeneic human mesenchymal stem cells for treatment of E. coli endotoxin-induced acute lung injury in the ex vivo perfused human lung. Proc Natl Acad Sci USA.

[55] Németh K., Leelahavanichkul A., Yuen P.S., Mayer B., Parmelee A., Doi K. (2009). Bone marrow stromal cells attenuate sepsis via prostaglandin E2–;dependent reprogramming of host macrophages to increase their interleukin-10 production. Nat Med.

[56] Leng Z., Zhu R., Hou W., Feng Y., Yang Y., Han Q. (2020). Transplantation of ACE2-mesenchymal stem cells improves the outcome of patients with COVID-19 pneumonia. Aging Dis.

[57] Shimabukuro-Vornhagen A., Gödel P., Subklewe M., Stemmler H.J., Schlößer H.A., Schlaak M. (2018). Cytokine release syndrome. J Immunother cancer.

[58] Wang H., Ma S. (2008). The cytokine storm and factors determining the sequence and severity of organ dysfunction in multiple organ dysfunction syndrome. Am J Emerg Med.

[59] Sadeghi S., Mosaffa N., Hashemi S.M., Naghizadeh M.M., Ghazanfari T. (2020). The immunomodulatory effects of mesenchymal stem cells on long term pulmonary complications in an animal model exposed to a sulfur mustard analog. Int Immunopharm.

[60] Pittenger M.F., Discher D.E., Péault B.M., Phinney D.G., Hare J.M., Caplan A.I. (2019). Mesenchymal stem cell perspective: cell biology to clinical progress. NPJ Regen Med.

[61] Lee K.-H., Tseng W.-C., Yang C.-Y., Tarng D.-C. (2019). The anti-inflammatory, anti-oxidative, and anti-apoptotic benefits of stem cells in acute ischemic kidney injury. Int J Mol Sci.

[62] Laffey J.G., Matthay M.A. (2017). Fifty years of research in ARDS. Cell-based therapy for acute respiratory distress syndrome. Biology and potential therapeutic value. Am J Respir Crit Care Med.

[63] Hu S., Li J., Xu X., Liu A., He H., Xu J. (2016). The hepatocyte growth factor-expressing character is required for mesenchymal stem cells to protect the lung injured by lipopolysaccharide in vivo. Stem Cell Res Ther.

[64] Harrell C.R., Sadikot R., Pascual J., Fellabaum C., Jankovic M.G., Jovicic N. (2019). Mesenchymal stem cell-based therapy of inflammatory lung diseases: current understanding and future perspectives. Stem Cell Int.

[65] Avanzini M.A., Mura M., Percivalle E., Bastaroli F., Croce S., Valsecchi C. (2021). Human mesenchymal stromal cells do not express ACE2 and TMPRSS2 and are not permissive to SARS-CoV-2 infection. Stem Cell Transl Med.

[66] Glenn J.D., Whartenby K.A. (2014). Mesenchymal stem cells: emerging mechanisms of immunomodulation and therapy. World J Stem Cell.

[67] Atluri S., Manchikanti L., Hirsch J.A. (2020). Expanded umbilical cord mesenchymal stem cells (UC-MSCs) as a therapeutic strategy in managing critically ill COVID-19 patients: the case for compassionate use. Pain Physician.

[68] Meng F., Xu R., Wang S., Xu Z., Zhang C., Li Y. (2020). Human umbilical cord-derived mesenchymal stem cell therapy in patients with COVID-19: a phase 1 clinical trial. Signal Transduct Targeted Ther.

[69] Nandina R.Q., Eriani K., Asrizal C.W., Azhar A. (2022). Mesenchymal stem cell as a successful therapy for COVID-19 patient: systematic review. Biosaintifika J Microbiol Biol Educ.

[70] Yu D., Li Y., Wang M., Gu J., Xu W., Cai H. (2022). Exosomes as a new frontier of cancer liquid biopsy. Mol Cancer.

[71] Modani S., Tomar D., Tangirala S., Sriram A., Mehra N.K., Kumar R. (2021). An updated review on exosomes: biosynthesis to clinical applications. J Drug Target.

[72] Dm P., Sj G. (2019). Exosomes. Annu Rev Biochem.

[73] Joo H.S., Suh J.H., Lee H.J., Bang E.S., Lee J.M. (2020). Current knowledge and future perspectives on mesenchymal stem cell-derived exosomes as a new therapeutic agent. Int J Mol Sci.

[74] Jafari D., Malih S., Eslami S.S., Jafari R., Darzi L., Tarighi P. (2019). The relationship between molecular content of mesenchymal stem cells derived exosomes and their potentials: opening the way for exosomes based therapeutics. Biochimie.

[75] Rodriguez A.-M., Nakhle J., Griessinger E., Vignais M.-L. (2018). Intercellular mitochondria trafficking highlighting the dual role of mesenchymal stem cells as both sensors and rescuers of tissue injury. Cell Cycle.

[76] Park J., Kim S., Lim H., Liu A., Hu S., Lee J. (2019). Therapeutic effects of human mesenchymal stem cell microvesicles in an ex vivo perfused human lung injured with severe E. coli pneumonia. Thorax.

[77] Vats D., Mukundan L., Odegaard J.I., Zhang L., Smith K.L., Morel C.R. (2006). Oxidative metabolism and PGC-1β attenuate macrophage-mediated inflammation. Cell Metabol.

[78] Phinney D.G., Di Giuseppe M., Njah J., Sala E., Shiva S., St Croix C.M. (2015). Mesenchymal stem cells use extracellular vesicles to outsource mitophagy and shuttle microRNAs. Nat Commun.

[79] Morrison T.J., Jackson M.V., Cunningham E.K., Kissenpfennig A., McAuley D.F., O’Kane C.M. (2017). Mesenchymal stromal cells modulate macrophages in clinically relevant lung injury models by extracellular vesicle mitochondrial transfer. Am J Respir Crit Care Med.

[80] Aliotta J.M., Pereira M., Wen S., Dooner M.S., Del Tatto M., Papa E. (2016). Exosomes induce and reverse monocrotaline-induced pulmonary hypertension in mice. Cardiovasc Res.

[81] Aliotta J.M., Pereira M., Wen S., Dooner M.S., Del Tatto M., Papa E. (2017). Bone marrow endothelial progenitor cells are the cellular mediators of pulmonary hypertension in the murine monocrotaline injury model. Stem Cell Transl Med.

[82] Song Y., Dou H., Li X., Zhao X., Li Y., Liu D. (2017). Exosomal miR-146a contributes to the enhanced therapeutic efficacy of interleukin-1β-primed mesenchymal stem cells against sepsis. Stem Cell.

[83] Willis G.R., Kourembanas S., Mitsialis S.A. (2017). Toward exosome-based therapeutics: isolation, heterogeneity, and fit-for-purpose potency. Front Cardiovasc Med.

[84] Braun R.K., Chetty C., Balasubramaniam V., Centanni R., Haraldsdottir K., Hematti P. (2018). Intraperitoneal injection of MSC-derived exosomes prevent experimental bronchopulmonary dysplasia. Biochem Biophys Res Commun.

[85] Khatri M., Richardson L.A., Meulia T. (2018). Mesenchymal stem cell-derived extracellular vesicles attenuate influenza virus-induced acute lung injury in a pig model. Stem Cell Res Ther.

[86] Gennai S., Monsel A., Hao Q., Park J., Matthay M., Lee J. (2015). Microvesicles derived from human mesenchymal stem cells restore alveolar fluid clearance in human lungs rejected for transplantation. Am J Transplant.

[87] Lee C., Mitsialis S.A., Aslam M., Vitali S.H., Vergadi E., Konstantinou G. (2012). Exosomes mediate the cytoprotective action of mesenchymal stromal cells on hypoxia-induced pulmonary hypertension. Circulation.

[88] Chen J-y, An R., Liu Z-j, Wang J-j, Chen S-z, Hong M-m (2014). Therapeutic effects of mesenchymal stem cell-derived microvesicles on pulmonary arterial hypertension in rats. Acta Pharmacol Sin.

[89] Hogan S.E., Rodriguez Salazar M.P., Cheadle J., Glenn R., Medrano C., Petersen T.H. (2019). Mesenchymal stromal cell-derived exosomes improve mitochondrial health in pulmonary arterial hypertension. Am J Physiol Lung Cell Mol Physiol.

[90] Yang K., Wang J., Wu M., Li M., Wang Y., Huang X. (2015). Mesenchymal stem cells detect and defend against gammaherpesvirus infection via the cGAS-STING pathway. Sci Rep.

[91] Meisel R., Zibert A., Laryea M., Göbel U., Da¨ubener W., Dilloo D. (2004). Human bone marrow stromal cells inhibit allogeneic T-cell responses by indoleamine 2, 3-dioxygenase–;mediated tryptophan degradation. Blood.

[92] Meisel R., Brockers S., Heseler K., Degistirici Ö., Bülle H., Woite C. (2011). Human but not murine multipotent mesenchymal stromal cells exhibit broad-spectrum antimicrobial effector function mediated by indoleamine 2, 3-dioxygenase. Leukemia.

[93] Thanunchai M., Hongeng S., Thitithanyanont A. (2015). Mesenchymal stromal cells and viral infection. Stem Cell Int.

[94] Bailey C.C., Zhong G., Huang I.-C., Farzan M. (2014). IFITM-family proteins: the cell’s first line of antiviral defense. Ann Rev Virol.

[95] Krasnodembskaya A., Song Y., Fang X., Gupta N., Serikov V., Lee J.-W. (2010). Antibacterial effect of human mesenchymal stem cells is mediated in part from secretion of the antimicrobial peptide LL-37. Stem Cell.

[96] Mei S.H., Haitsma J.J., Dos Santos C.C., Deng Y., Lai P.F., Slutsky A.S. (2010). Mesenchymal stem cells reduce inflammation while enhancing bacterial clearance and improving survival in sepsis. Am J Respir Crit Care Med.

[97] Oliphant S., Lines J.L., Hollifield M.L., Garvy B.A. (2015). Regulatory T cells are critical for clearing influenza A virus in neonatal mice. Viral Immunol.

[98] Rao L., Xia S., Xu W., Tian R., Yu G., Gu C. (2020). Decoy nanoparticles protect against COVID-19 by concurrently adsorbing viruses and inflammatory cytokines. Proc Natl Acad Sci USA.

[99] Zhang H., Penninger J.M., Li Y., Zhong N., Slutsky A.S. (2020). Angiotensin-converting enzyme 2 (ACE2) as a SARS-CoV-2 receptor: molecular mechanisms and potential therapeutic target. Intensive Care Med.

[100] Zhang Q., Honko A., Zhou J., Gong H., Downs S.N., Vasquez J.H. (2020). Cellular nanosponges inhibit SARS-CoV-2 infectivity. Nano Lett.

[101] Leeming D., Genovese F., Sand J., Rasmussen D., Christiansen C., Jenkins G. (2021). Can biomarkers of extracellular matrix remodelling and wound healing be used to identify high risk patients infected with SARS-CoV-2?: lessons learned from pulmonary fibrosis. Respir Res.

[102] Hosseini N.F., Dalirfardouei R., Aliramaei M.R., Najafi R. (2022). Stem cells or their exosomes: which is preferred in COVID-19 treatment?. Biotechnol Lett.

[103] Raghav A., Khan Z.A., Upadhayay V.K., Tripathi P., Gautam K.A., Mishra B.K. (2021). Mesenchymal stem cell-derived exosomes exhibit promising potential for treating SARS-CoV-2-infected patients. Cells.

[104] Fujita Y., Kadota T., Araya J., Ochiya T., Kuwano K. (2018). Clinical application of mesenchymal stem cell-derived extracellular vesicle-based therapeutics for inflammatory lung diseases. J Clin Med.

[105] Li D.-Y., Li R.-F., Sun D.-X., Pu D.-D., Zhang Y.-H. (2021). Mesenchymal stem cell therapy in pulmonary fibrosis: a meta-analysis of preclinical studies. Stem Cell Res Ther.

[106] Dinh P.-U.C., Paudel D., Brochu H., Popowski K.D., Gracieux M.C., Cores J. (2020). Inhalation of lung spheroid cell secretome and exosomes promotes lung repair in pulmonary fibrosis. Nat Commun.

[107] Glassberg M.K., Minkiewicz J., Toonkel R.L., Simonet E.S., Rubio G.A., DiFede D. (2017). Allogeneic human mesenchymal stem cells in patients with idiopathic pulmonary fibrosis via intravenous delivery (AETHER): a phase I safety clinical trial. Chest.

[108] Metcalfe S.M. (2020). Mesenchymal stem cells and management of COVID-19 pneumonia. Medicine Drug Discov.

[109] Abdelgawad M., Bakry N.S., Farghali A.A., Abdel-Latif A., Lotfy A. (2021). Mesenchymal stem cell-based therapy and exosomes in COVID-19: current trends and prospects. Stem Cell Res Ther.

[110] Csobonyeiova M., Smolinska V., Harsanyi S., Ivantysyn M., Klein M. (2023). The immunomodulatory role of cell-free approaches in SARS-CoV-2-induced cytokine storm—a powerful therapeutic tool for COVID-19 patients. Biomedicines.

[111] Aljuhani A., Albalawi O., Albalawi R., Alsalama R., Alatawi S., Altemani R. (2023). Exosomes in COVID-19 infection: focus on role in diagnosis, pathogenesis, immunity, and clinical trials. Cell Biol Int.

[112] Singh C.K., Sodhi K.K. (2023). The emerging significance of nanomedicine-based approaches to fighting COVID-19 variants of concern: a perspective on the nanotechnology’s role in COVID-19 diagnosis and treatment. Front Nanotechnol.

[113] Chakravarty M., Vora A. (2021). Nanotechnology-based antiviral therapeutics. Drug Deliv Transl Res.

[114] Abbaspour-Aghdam S., Hazrati A., Abdolmohammadi-Vahid S., Tahmasebi S., Mohseni J., Valizadeh H. (2022). Immunomodulatory role of Nanocurcumin in COVID-19 patients with dropped natural killer cells frequency and function. Eur J Pharmacol.

[115] Martín Giménez V.M., Prado N., Diez E., Manucha W., Reiter R.J. (2020). New proposal involving nanoformulated melatonin targeted to the mitochondria as a potential COVID-19 treatment. Nanomedicine.

[116] Wu J., Wang H., Li B. (2020). Structure-aided ACEI-capped remdesivir-loaded novel PLGA nanoparticles: toward a computational simulation design for anti-SARS-CoV-2 therapy. Phys Chem Chem Phys.

[117] Tousian B., Khosravi A.R. (2023). Chitosan-based pulmonary particulate systems for anti-cancer and antiviral drug carriers: a promising delivery for COVID-19 vaccines. Results Chem.

[118] Rasul R.M., Muniandy M.T., Zakaria Z., Shah K., Chee C.F., Dabbagh A. (2020). A review on chitosan and its development as pulmonary particulate anti-infective and anti-cancer drug carriers. Carbohydr Polym.

[119] Mehta M., Prasher P., Sharma M., Shastri M.D., Khurana N., Vyas M. (2020). Advanced drug delivery systems can assist in targeting coronavirus disease (COVID-19): a hypothesis. Med Hypotheses.

[120] Valizadeh H., Abdolmohammadi-Vahid S., Danshina S., Gencer M.Z., Ammari A., Sadeghi A. (2020). Nano-curcumin therapy, a promising method in modulating inflammatory cytokines in COVID-19 patients. Int Immunopharm.

[121] Meng Q.-F., Tai W., Tian M., Zhuang X., Pan Y., Lai J. (2023). Inhalation delivery of dexamethasone with iSEND nanoparticles attenuates the COVID-19 cytokine storm in mice and nonhuman primates. Sci Adv.

[122] Chen L., Qu J., Kalyani F.S., Zhang Q., Fan L., Fang Y. (2022). Mesenchymal stem cell-based treatments for COVID-19: status and future perspectives for clinical applications. Cell Mol Life Sci.

[123] Choudhery M.S., Harris D.T. (2020). Stem cell therapy for COVID-19: possibilities and challenges. Cell Biol Int.

[124] Li C., Zhao H., Wang B. (2020). Challenges for mesenchymal stem cell-based therapy for COVID-19. Drug Des Dev Ther.

[125] Ghiasi M., Jadidi K., Hashemi M., Zare H., Salimi A., Aghamollaei H. (2021). Application of mesenchymal stem cells in corneal regeneration. Tissue Cell.

[126] Missoum A. (2020). Recent updates on mesenchymal stem cell based therapy for acute renal failure. Curr Urol.

[127] Qian X., Xu C., Fang S., Zhao P., Wang Y., Liu H. (2016). Exosomal microRNAs derived from umbilical mesenchymal stem cells inhibit hepatitis C virus infection. Stem Cell Transl Med.

[128] Golchin A. (2021). Cell-based therapy for severe COVID-19 patients: clinical trials and cost-utility. Stem Cell Rev Rep.

[129] Li S., Zhu H., Zhao M., Liu W., Wang L., Zhu B. (2022). When stem cells meet COVID-19: recent advances, challenges and future perspectives. Stem Cell Res Ther.

[130] Yeo R.W.Y., Lai R.C., Zhang B., Tan S.S., Yin Y., Teh B.J. (2013). Mesenchymal stem cell: an efficient mass producer of exosomes for drug delivery. Adv Drug Deliv Rev.

[131] Mendt M., Kamerkar S., Sugimoto H., McAndrews K.M., Wu C.-C., Gagea M. (2018). Generation and testing of clinical-grade exosomes for pancreatic cancer. JCI Insight.

[132] Andriolo G., Provasi E., Lo Cicero V., Brambilla A., Soncin S., Torre T. (2018). Exosomes from human cardiac progenitor cells for therapeutic applications: development of a GMP-grade manufacturing method. Front Physiol.

[133] Yang D., Zhang W., Zhang H., Zhang F., Chen L., Ma L. (2020). Progress, opportunity, and perspective on exosome isolation-efforts for efficient exosome-based theranostics. Theranostics.

